# Perceptions of Ecuadorian indigenous healers on their relationship with the formal health care system: barriers and opportunities

**DOI:** 10.1186/s12906-021-03234-0

**Published:** 2021-02-18

**Authors:** Estefanía Bautista-Valarezo, Víctor Duque, Veronique Verhoeven, Jorge Mejia Chicaiza, Kristin Hendrickx, Ruth Maldonado-Rengel, Nele R.M. Michels

**Affiliations:** 1 Departamento de Ciencias de la Salud, Universidad Técnica Particular de Loja (UTPL), San Cayetano alto s/n, 1101608 Loja, CP Ecuador; 2 Department of Primary and Interdisciplinary Care, Faculty of Medicine and Health Sciences, University of Antwerp, Universiteitsplein 1, 2610 Antwerp, Belgium; 3 Universidad de Cuenca, Cuenca, Ecuador

**Keywords:** Latin America, Indigenous, Ecuador, Cultural skills, Relationship, Intercultural health and traditional healers

## Abstract

**Background:**

The new paradigm of intercultural policies focuses on rethinking the common public culture. In Ecuador, the “*Buen Vivir*” plan seeks to incorporate the ancestral medical knowledge, experience and beliefs of traditional healers into the formal health services. This study explores views on the formal health system from the perspective of the healers belonging to the Kichwa and Shuar ethnicities in the South of Ecuador.

**Methods:**

A qualitative study with a phenomenological approach was performed. Focus groups were conducted in three locations in Southern Ecuador. Shuar, Kichwa and Mestizo ethnic groups were included in the research.

**Results:**

Eleven focus groups with a total of 110 participants belonging to the Shuar, Kichwa and Mestizo ethnic groups participated in the study. Six themes were created through analysis: 1) conflicts with health professionals, 2) acceptance of traditional healers, 3) respect, 4) work as a team, 5) environment and patient care, and 6) salary and recognition.

**Conclusion:**

This study indicated the perceived barriers compromising respectful collaboration between health staff and traditional healers from an indigenous perspective. Power inequalities and a historically unidirectional relationship and, in addition, differences in health beliefs, seem to create misunderstandings regarding each other’s approach when faced with health and disease. However, insight in these barriers can create opportunities towards collaboration, which will have a positive effect on patient confidence in one or both systems and support continuity between traditional healers and the formal health system.

## Background

Currently, in Latin America, interculturality is born from the struggle of indigenous organizations that seek recognition, equality, respect and inclusion, mainly in areas of health and education [1]. This paradigm establishes interculturality as a tool to support the consolidation of a more equitable and participatory social system [2, 3].

The 1998 Constitution defined the Ecuadorian state as multicultural, multiethnic, and which incorporated “traditional medicine” into the National Health System [4]. This concept was strengthened in the 2008 *“Montecristi”* Constitution by creating laws, projects, and plans that regulated indigenous participation with the State [5]. From these constitutions, the organic Health Law (2006) was born, which is in force to date. In chapter II, the law proposes “the promotion, implementation, and control of traditional medicine practices”. Besides, it promotes knowledge exchange between the different health system actors and healers [6].

Interculturality is established as a tool supporting the consolidation of a more equitable and participatory social system. There is a new paradigm of intercultural politics focusing on the cohesion and rethinking of a common public culture [7]. In 2009, Ecuador adopted a new policy through the “*Buen Vivir”* National Plan or “Sumak Kawsay”. In the domain of Medicine, the plan seeks to integrate the experience and beliefs of traditional healers with the formal, biomedical, health services [8, 9]. A clear implementation National Plan is the cultural adaptation of delivery rooms, where vertical delivery care is a reality for indigenous women [10].

Through the “*Buen Vivir*” national plan, the government recognized the role of traditional healers in primary care. This recognition clearly supports a better climate for traditional healers and health staff to work together. It is known that healers and biomedical health staff have differences in treatments, diagnoses and conceptions of what health and illness means. These differences can create barriers that negatively influence collaboration [8, 9].

For the health care system, it is primordial to recognize these barriers and turn them into opportunities. In order to do that, it is essential to have good insights on 1) how traditional healers interpret the concepts of health and illness, and 2) which barriers healers perceive for health staff and healers to work together.

For the first question, in a previous article we described the Ecuadorian indigenous healers’ perceptions about health and illness. Through a qualitative study we found that views on health and illness differ from the Western biomedical care model. For example, the indigenous perspective of health and illness focuses on a balance between 4 bodies: the physical, spiritual, social, and mental bodies. Additionally, “good health” is attained through a good diet and balance/harmony [11].

The second question, which is looking for ways of collaboration, was previously studied by some authors. In a South African study, healers recognize the need to work as a team to improve the health of their communities. However, bad relationships between healers and medical staff is still noticed [12, 13]. In 1992, Hoff studied the work of traditional healers in primary care teams. The author refers to some constrains like 1) absence of clear recognition, 2) lack of dialogue, and 3) unclear role of the healer in relation to other members of the primary care team [14].

Therefore, and related to a project in which we study the needs and the conditions that compromise intercultural health care in the Kichwa and Shuar communities in Ecuador, we feel the need to explore, from an indigenous perspective, the barriers that compromise the relationship between healers and health staff. In addition, we want to define facilitators and look at opportunities for collaboration. In this way, we can provide concrete measures of effective implementation of an intercultural health policy and implementation of intercultural integration as well as for practitioners in the field, and for students in the medical curriculum [15].

## Methods

### Research design

A qualitative observational study was performed over the period of one year in Southern Ecuador, in the region of Saraguro, Yacuambi and Cuenca (Senplades, Zones 6 and 7)] [16]. We seek to deepen our understanding of opinions and perceptions of the healers belonging to the Kichwa and Shuar ethnicities. The performance of focus groups was chosen to collect data because this method allows the participants to interact with each other in a comfortable environment, and we wanted to establish what the people really think and feel [17].

### Ethical approval

The ethical requirements of the Research Committee of San Francisco de Quito University were met, and they approved the study (CEISH USFQ 2017-059E). Written consent was obtained from the indigenous community (the “Saraguro y Cuenca” Healers Council) and participants, who were free to decline participation or withdraw at any time.

### Recruitment and setting

We used a convenience sampling strategy using volunteer and purposive methods. All participants were selected specifically because they had some experience of interest to the study and they are healers recognized by the communities. To facilitate discussions on diverse levels, different types of healers were recruited, such as: a) midwifes *(parteras)* b) shaman or uwishin, c) bonesetters *(sobadores)* and d) herb-healers *(hierbateros).* The inclusion criteria were the following: working as a ‘traditional’ healer or midwife *(partera)* and having the ability to speak Spanish. However, the participants’ mother language was not Spanish, which explains the grammatical errors that can be found in the quotes. We chose to recruit indigenous groups from Southern Ecuador (Saraguros, Shuar and Cholos Cuencanos) because a) these communities are the center of meetings for indigenous celebrations, and b) these communities belong to native Ecuadorian people.

### Data collection

The focus groups started with an initial orientation (aims of the study) and engagement with the participants. Additionally, informed consent and permission to audio-record the discussions were requested. The participants were ensured confidentiality of their contributions. The focus groups were conducted with the help of two moderators, and two observers, who made field notes [18]. The focus group interview guide consisted of questions focused on traditional healers’ views on the public health policies, the relationships with other health staff and the perceived barriers.

In this article we analyzed answers on the last two questions related to views on formal health systems (Table 1).

**Table 1:** Focus group interview guide

**Health and illness process:** What does it mean for you to be healthy?	
What does it mean for you to be sick?	
What should people do to stay healthy?	
What should people do to heal themselves?	
**Diagnostic and treatment resources and practices:** What resources do you use to diagnose diseases?	
What resources are used to heal?	
**Views on formal systems:** What do you think about physicians?	
When do you send a patient to the Western physician/Health system?	

### Data analysis

Thematic content analysis with a phenomenological approach was developed as an interpretive base of the focus group. This means that our analysis seeks to explore the perceptions, opinions and interpretations that participants make about the research question. It also involves contrasting data from different perspectives and data interpretation.

The recorded focus group data and observational notes were literally transcribed and analyzed by using N-Vivo software. Every focus group was analyzed separately and important quotes were coded and categorized in themes and sub-themes to form a code book. The code book was shared among three researchers who collectively decided on the description of the quotes, until all codes were extracted. The quotes were clustered and connected under broader themes trying to identify core variables that emerge in a new theory or explanation model [19–21].

Code saturation was achieved in the fourth focus group, the rest of the focus groups was still analyzed until meaning saturation was reached in all codes [22]. The quotes that best represented themes and sub-themes were incorporated into the analysis.

## Results

The final sample comprised 110 participants aged 20–70 years old. The participants in the study were the healers from the region of Saraguro, Yacuambi and Cuenca, working as shamans (“yachay” / “uwishin” or “*visionarios”),* herb-healer (“*hierbateros”),* midwife *(“parteras”),* or bonesetter (*“sobadores*”). The parity of participants included members of the Shuar, Kichwa (Cholos Cuencanos and Saraguros) or Mestizo ethnicities, which are the ethnic groups living in Senplades zones 6 and 7 in Southern Ecuador (Table 2). Eleven focus groups were conducted with different participants from different ethnicities. The focus groups lasted between 98 and 167 min, plus 30 min for initial orientation and engagement with the participants (Table 3).

**Table 2:** Sociodemographic characteristics

Characteristic	N (%)
**Gender**	
Female	80 (72,7)
Male	30 (27,3)
**Age (years)**	
< 30	17 (15,4)
30–49	51 (46,4)
50–69	39 (35,5)
> 70	3 (2,7)
**Ethnia**	
Kichwa	88 (80)
Mestizo	2 (1,8)
Shuar	20 (18,2)
**Education**	
Illiterate	11 (10)
Primary	45 (40,1)
High school	51 (46,4)
Higher	3 (2,7)
**Healer**	
Yachay / Uwishin or Visionaries	10 (9,1)
Midwife *(“Parteras”)*	65 (59,1)
Herb-healer *(“Hierbateros”)*	26 (23,6)
Bonesetter *(“Sobadores”).*	9 (8,2)

**Table 3:** Focus Groups

Identification Code	Date	Place	Ethnicitis^a^	Number of participants
FG1	18/08/16	Saraguro	Kic, M	10
FG2	17/08/16	Yacuambi	Sh, Kic	10
FG3	18/08/16	Saraguro	Kic, M	10
FG4	18/08/16	Saraguro	Kic, M	10
FG5	17/08/16	Yacuambi	Sh, Kic,	10
FG6	08/08/16	Saraguro	Kic, M	9
FG7	17/08/16	Yacuambi	Sh, Kic,	10
FG8	07/08/16	Yacuambi	Sh, Kic,	10
FG9	12/10/16	Cuenca	Kic, M	10
FG10	13/10/16	Cuenca	Kic	10
FG11	25/10/16	Cuenca	Kic, M	11

^a^Ethnia: Kic: Kichwa, M:Mestizos, Sh:Shuar

In general, the analysis of the focus groups reveals that traditional, ancestral medicine is a kind of healing practice aimed at improving or maintaining the health of the individuals and of the communities. Traditional healers, carry it out using their ancestral knowledge and skills. Usually, this ancestral medicine is practiced outside the formal health system. However, traditional healers seek complementarity with the formal health system. In their eyes, both paradigms of healing should coexist in favor of the patient.

In addition, more specifically, the analysis shows that the healers’ views on the health system can be categorized into six main sub-themes (Table 4).

**Table 4:** Sub-themes

***Views on the formal health system***	
*Conflicts with health professionals*	
*Acceptance of traditional healers*	
*Respect*	
*Work as a team*	
*Environment and patient care*	
*Salary and recognition*	

In the next paragraphs, the six subtopics will be described.

### Conflicts with health professionals

The relationship between traditional healers and health staff clearly has a perspective of power. It is strongly marked by the health professionals’ domination to the detriment of traditional healers (Q1).

*Q1-FG6: “One is thrown out, because he or she is not wearing white clothes [medical gown], the opinion of one does not assert. I do not know why..., but one is humiliated, we deserve respect, each one with its profession.” Kichua/woman*

Physicians are seen as arrogant people that really believe they know everything. They are incapable to understand what the traditional healer can contribute because of their lack of knowledge of ancestral medicine and skills (Q2-Q3). They criticize the work the healers do for the improvement of the community and the individuals’ health and impose their authority on healers (Q4). Moreover, they plan therapeutic solutions as orders, without consensus or more culturally appropriate alternatives (Q5). The healers do not support the health professionals attempt to establish superiority over them and imagine a scenario where both can work as equals, each one with their abilities and knowledge.

According to the data, some factors emerge as responsible for the healers’ subordinate status and others are used to empower themselves and their social importance.

*Q2-FG1: “In the head of the doctors there is still no interculturality. Let’s say, it is only in the word but nothing more, according to them they totally know everything. Of course, we are there, but they do not take us into account.” Kichua/man*

*Q3-FG1: “They do not value our medicine; they do not know our skills and do not value it.” Shuar/man*

*Q4-FG8: I first sent him [patient] to take an X-ray before I treat him and after too. But, the traumatologist tells him that “you've already gone to a bonesetter that is of no use”..., I would never have said that. I send the X-ray to see how it comes and how it has remained.” Kichua/man*

*Q5-FG1: “Sometimes the “white coat” want to rule by pushing to a poor person.” Kichua/woman*

For one particular term positive as well as negative connotations are used; the “countryside” is, on the one hand, associated with ignorance and humbleness and also with a servile attitude (Q6). Healers consider their rural reality to be the cause of contempt and loss of opportunity (Q7). On the other hand, the “countryside” is named as something positive and empowering when it is associated with nature and resources. For example, it relates to their territory, where they are more needed and people come to them. The “field” certainly becomes a positive factor when used as a quality of medicine or food (field countryside, field medicine).

*Q6-FG1: “and me, because of that humbleness, as one is from the countryside, we even are afraid to speak with them.” Kichua/woman*

*Q7-FG1: “we did not have the opportunity to work as doctors, that is, we were from the field.” Kichua/woman*

According to the healers, physicians derive their superiority from their educational process (higher education) (Q8). “Having studied” is seen as powerful but also as the seed for their superior behavior and the contempt for healers (Q9). Sometimes they assume that some errors or misunderstanding of the health professionals may be the result of “lack of study”. Healers oppose the medical knowledge that comes from the study with their own knowledge that comes from the experience and from their ancestors. Healers think that both methods can complement each other (Q10-Q11).

*Q8-FG6: “We always had to be rejected. They say that we are empirical and doctors have studied and we do not have the right to do medicine.” Kichua/man*

*Q9-FG4: “There are some doctors who do know, who have studied, but do not respect us, tell us your medicine is bad and you do not understand that pills and blisters are our hands.” Kichua/woman*

*Q10-FG3: “As a mother, as an old woman... I speak to the young ladies’ nurses or the doctors, I say... you know for the study and I know for giving birth to many children and raising them. That is my experience of sharing with you.” Kichua/woman*

*Q11-FG3: “Maybe we don’t talk by science but we talk by experience [...] what we lived, what we felt, the daily practice… that has made us grow.” Shuar/man*

### Acceptance of traditional healers

The healers claim acceptance. They say that they accept the western medicine and trust the health professionals, but they need also to be accepted as well to build a collaborative relationship (Q12). They also mention that acceptance from the health professionals makes them feel better. Acceptance is understood in several senses. On the one hand, as the acceptance of their own diseases, their own understanding of health and illness, and their entire worldview. On the other hand, as the acceptance of the healer’s existence and their role in society. The health staff seems to be restricted to the epistemological framework they learnt at the university.

However, they feel an improvement of the situation and believe that this is related with the new constitution and laws and with the fact that indigenous people make themselves heard (Q13–14).

*Q12FG1: “Well, for us, we do not... as healers we have that... that too much trust... that too much security that we are good with them [physicians], but instead they are the opposite with us.” Kichua/man*

*Q13-FG5: “since I started there was discrimination. Then, with the constitution it has changed...” Kichua/woman*

*Q14-FG1: “In any case we have discussed with them, but, I have not kept quiet. Now, lately the nurses have accepted me, right now everything I do. [...] they no longer argue. Now, there is more confidence, but, earlier not, and I had to argue a lot with them, fight a lot.” Kichua/man*

Even though indigenous healers say that they accept the formal system and its professionals and that they do not show a total confrontation or rejection of the formal system, they also request more information about the treatment process and possibilities and they do not blindly believe that what the doctors say is the truth or the only possible solution (Q15). They want to discuss with physicians the different possibilities and approaches, taking into account the cultural differences and the healers’ healing skills (Q16).

*Q15-FG3: “I was eight days in the hospital, it is bittersweet to be in a hospital bed. One pill a day and the doctors come and say: “are you okay? How are you responding?”, and a tap and “we visit you tomorrow”. Finally, I said, "Are you going to attend to me or what is going on?" "No," he said, "it's already convalescing." [...] I said “if you will not attend me, then I go”. He replied “you do not know that we have to put some nails, we will operate, if it heals well if not…” My knee, it is not wood, it is not plaster, it is flesh and bone! “Please, I do not want anything” - “Sir, then, if you do not want any of that, go away” I said “thank you”, I told him that I will come back someday and I left in the elevator.” Kichua/woman*

*Q16-FG4:* ***“****The doctors do not value our medicine, the ministry tells us to come to the hospital, but when we arrive the nurses treat us badly” Kichua/man*

### Respect

The narratives about the relationship are fraught with references to the need for respect from physicians (Q17). Respect is seen as a way for peaceful coexistence and is also necessary for teamwork. According to healers, the physicians just claim for respect but without returning respect in the same way.

*Q17-FG1: “It would be nice if the doctors recognized us... if they respected our opinions, our decisions, our knowledge... that when we went to the hospital, we were treated well. It would be excellent to work like this, but if they respected us, our ideology, everything; it would be nice. But there is still no such thing”. Kichua/woman*

### Work as a team

The indigenous healers believe that the formal health system is incapable to attend to all population needs. It lacks knowledge about some indigenous realities and diseases, and it cannot diagnose and treat them (Q18). They also consider that intercultural health means a joint approach by healers and physicians.

*Q18-FG6: “The latest technology does not find a “Shuca”, but in the field we can see that.” Kichua/woman*

Neither approach can fully address the health problems of the population. The opportunity to complement each other clinically and culturally will allow to build bridges of intercultural collaboration for the patient and a better health system.

Due to a lot of reasons healers think that working as a team is the best way to improve the health of their communities. To constitute a good team, it is important to have mutual recognition and that both parts shares, are been seen by each other, and have attributions (Q19).

*Q19-FG6: “We believe it would be good to work as a team… .to be able to heal people and take care of the health of the community.” Kichua/man*

### Environment and patient care

In the healers’ narratives, the hospital constantly appears as a hostile environment (Q20), both for a healer as for a patient. It is seen as a place where patients are not cared properly, which makes them embarrassed, humiliated, and afraid of being mistreated. Their clothes are removed and changed; as clothes are an important identity symbol, they feel left naked. Another key factor is the food. They think that the hospital food is detrimental to health and make people weaker. Some hospital practices, such as taking showers for the women who have just given birth, are against the common rituals of the indigenous people and they think that they make women sick. Hospitals are also cold places, which have an enormous importance in their health cosmovision, based on warm-cold systems where cold usually means illness.

Q20-FG4: *“With the bad experiences I've had, that is, going to the hospital for me is having anger.” Kichua/woman*

Furthermore, indigenous people are afraid of some western treatments, mainly surgeries. They think that physicians do it even if it is not the only solution and without first trying other ways to solve the problem (Q21). They relate surgeries to long-term complications that prevent them from working (Q22). In addition to that, they do not feel sufficiently informed by health professionals. There are also some references to medical abandonment when someone does not follow their instructions or disagrees with something.

*Q21-FG2: “They told me that they were going to operate because it was already in abscess, but it was not yet, so I had to flee from there, the tumor was not developed and we lowered that swelling. That is a negative part that it was going to be removed and do not…” Kichua/woman*

*Q22-FG1: “For us as peasants, as women, is not a solution [the Caesarean section], of course that is going to solve the case, but then there will be complications. We need to work in the field, we want to make efforts, we need to be complete, we do not need to be broken, rather, in that sense there is no such saying ... that desire to want our knowledge.” Kichua/woman*

Despite the aforementioned arguments, there are also reasons to go to a hospital: healers can see that hospitals have more possibilities and fewer bureaucratic problems (Q23). Especially the midwives or “*parteras”* mostly agree with this, nevertheless, they prefer to go to a hospital which has “culturally adapted delivery rooms”. In these rooms, they are allowed to prepare their own food, to use medical plants and to stay with the family. Besides, if it is needed the physicians can be called quickly (Q24).

*Q23-FG8: “We want our opinions to be respected when we take a patient to the hospital. I am working for 6 months in the hospital and now I have been accepted in the hospital.” Kichua/woman*

*Q24-FG8: “I send all of my patients to the hospital for a vertical delivery because there is good care. They let them cook, eat the chicken criolla because usually in the hospital they give things that a parturient should not eat .. ., and they didn't like it..” Kichua/woman*

Some participants mention that, when they send their own patients to hospitals or physicians, they lose control and the possibilities to care of them, as if they abandon the patients (Q25). In addition to that, people do not want to go alone to the physician because they are afraid (Q26). For them a solution should be to allow healers to accompany the patient.

*Q25-FG2: “if I say to my patients: "go to the hospital" I can no longer attend them.” Kichua/woman*

*Q26-FG3: “We, when they already pray to me, I'm going to see, they tell me, we're going to stay with them and we're going to visit, then, we go and sometimes they do not want to go back in fear, they say they're not cool they're cold.” Kichua/woman*

### Salary and recognition

Historically, healers where reasserted by their communities, they believe that the statements of the people whom they have healed is the best proof of their skills and it is also the reason why more people keep coming to be healed.

At this time, they feel that a governmental certification is needed. Persecutions in the past for practicing ancestral medicine, the abuses they receive from physicians, requirements of the new laws and the emergence of false healers (“charlatans” and “scammers”) are responsible for this request/need (Q27). According to the healers, this support would allow them to do their work with better communication and cooperation with doctors and nurses and would contribute to higher acceptance, even inside hospitals and health centers; at the same time, it would give them more confidence to actively participate. They are aware of the internal difference among healers but, despite that, they believe that a certification will help them fight against the distrust, while it is not important for the service they offer (Q28).

*Q27-FG1: “We midwives do not receive support, the ministry tells us to come to pay attention here in the hospital, we fix a place, but they do not give us a bonus, I can attend for free, I have a family to feed.” Kichua/woman*

*Q28-FG6: “I would say that I do not need a card or a certificate because that will not heal. We heal with our hands. But maybe it will be needed to be identified and that could be respect.” Shuar/man*

In some interventions they also claim for an economic consideration, mainly the midwives who have to move to the intercultural delivery rooms by their own means (Q29). Some emphasize that the what matters is the service, not the money, and they assume this as a difference with the false healers who only want to be rich, which is seen as selfish and morbid. Because of that, traditional healers used to ask for voluntary payment and they do not charge anything if individuals do not have money. However, they think that it is important to have some payment in order to value the ancestral medicine.

*Q29-GF1: “The nurses make money, but, they do not care ... They went to sleep and another healing partner who is there attending to another person because he has begged…” Kichua/woman*

## Discussion

The described experiences and perceptions of indigenous healers highlight the current barriers compromising better collaboration between healers and biomedical health professionals in the South of Ecuador. Besides, they provide a starting point to take advantage of the opportunities ahead.

The last three decades there was a change in the government’s policies and laws in South America.

Since 2009 in Ecuador a new regimen works under guidelines of interculturality, equity and a new social and sustainable development, called *“Buen vivir*” [7, 8]. The principal evidence of this framework is the presence of interculturality as an important axis in health. Nevertheless, the lack of an operative model or management model in order to implement this intercultural approach in the health system makes it difficult for the stakeholders in the field to bring these ideas into practice [23].

Various obstacles appear for both health professionals and indigenous healers to build a better relationship that will allow a coordinated service for the patient. Power inequalities and a historically unidirectional relationship between health staff and healers compromise a respectful collaboration. This is not only linked with ethnical differences but also with the rural context. Another problem is that differences in health beliefs make that both parties do not always understand each other’s approach when confronted with illness. Mutual criticism between healers and health professionals undermines patients’ trust in one or both systems and hamper continuity of care between healers and the formal system [12, 14, 24].

In addition to our findings, some other barriers are described in literature. First, there seem to be a lack of experienced health staff in rural areas in Ecuador; the physicians are often newly graduated and stay just for one year to achieve their work permit in the country. Second, the legal framework in which both parties operate is not very well known and it has changed a lot in the last years; bureaucratic barriers create fear and uncertainty in healers when they have to undertake action [25].

Our research was conducted with the ultimate goal to improve the intercultural relationship between healers and health staff. This article provides the encouragement for the implementation of intercultural plans, both among rural health professionals and in the medical curriculum. In this context, the barriers that came up in our study are seen as challenges and opportunities to create the preconditions needed for successful collaboration.

Also, in Chile Camila Pérez identified some factors supporting the collaboration between medical staff and healers of the Mapuche population: “laws and regulations pertaining to the rights of indigenous peoples, the empowerment of users around their rights, the formation of implementation teams, the presence of professionals of Mapuche origin in health facilities, and the existence of processes for the systematization of the work carried out” [26].

Based on our research, we propose the following recommendations to facilitate intercultural collaboration:

1) The government can help with laws or programs that actively involve indigenous healers in a care team, especially in rural health. Also, the strengthening of technical areas (National Directorate of Interculturality, Rights, and Social Participation) will allow an effective implementation and a better understanding of the community’s health status and create bridges of empathy among all those involved. 2) The healers themselves will also need to contemplate their role in the perceived problems. Both groups need to broaden their minds and be open to collaboration. 3) The academies and universities should support the development of intercultural competencies in medical studies. As such, medical students will learn about values ​​and human rights that allow them to enter and reflect on the complexity of each individual, culture and people; 4) Finally, a “knowledge dialog”, in which health staff and healers practice skills together and teach each other, could be organized. As a good practice, we can refer to the Private Technical University of Loja, Ecuador (UTPL, *Universidad Técnica Particular de Loja*) [16, 27].

A limitation of this study is that it only collects information from focus groups and a deeper analysis would be expected with interviews. Furthermore, we only studied the healer’s perspective and, besides, the difference between the cosmovisions of the Shuar and Kichwa nationalities was not investigated, despite the fact that the data allows us to presume that some important differences exist.

On the other hand, strengths include the continuous relationship between the research team and the healers, which allows the latter to talk in confidence and comfort and the researchers to better interpret the results. The profile and diversity of researchers can be stressed: the researchers come from three different universities and three different countries, and some of them have worked with indigenous communities in the past. We believe that this has enriched the analysis of the collected data.

In order to move on with improving collaboration between healers and health staff in the rural areas of Ecuador, we plan some further research. Ways of structured collaboration will be developed focusing on some specific health issues such as fever in children and obstetric emergencies.

## Conclusion

The recognition of traditional medicine in the health system through policies and laws was achieved by the hard work of indigenous groups. These laws aim at equality and inclusion; however, healers, who have experience with working together with a health professional, express their dissatisfaction with power inequalities, lack of recognition and physician’s narrow-mindedness.

This study reveals the main points of the lack of legal mechanisms for recognizing the role of healers in the health system. It recommends a more effective implementation of intercultural health policies, and a reflection of both health staff and healers on their contribution in the collaboration Furthermore, a training in intercultural skills for medical students and all healthcare workers is necessary to create an atmosphere of respect, equity, and understanding.

## Data Availability

The datasets used and analyzed during the current study are available from the corresponding author upon reasonable request.

## Abbreviations

CEISH USFQ: The Research Committee of the San Francisco de Quito University
Kic: Kichwa
Sh: Shuar
M: Mestizos
